# Is Borg’s perceived exertion scale a useful indicator of muscular and cardiovascular load in blue-collar workers with lifting tasks? A cross-sectional workplace study

**DOI:** 10.1007/s00421-013-2782-9

**Published:** 2013-12-13

**Authors:** Markus Due Jakobsen, Emil Sundstrup, Roger Persson, Christoffer H. Andersen, Lars L. Andersen

**Affiliations:** 1 National Research Centre for the Working Environment, Lersø Parkalle 105, 2100 Copenhagen, Denmark; 2 Institute of Sports Science and Clinical Biomechanics, University of Southern Denmark, Odense, Denmark; 3 Department of Psychology, Lund University, Lund, Sweden

**Keywords:** Borg, EMG, Heart rate, Self-report, Pain

## Abstract

**Purpose:**

To investigate associations between perceived exertion and objectively assessed muscular and cardiovascular load during a full working day among workers with manual lifting tasks.

**Methods:**

A total of 159 men and 41 women from 14 workplaces with manual lifting tasks participated. Participants reported perceived exertion (BORG-CR10) at midday and after work. Surface electromyography of the thigh, lower back and neck muscles were normalized to isometric voluntary contractions (MVC) to express relative muscle load during the day. Cardiovascular load was measured with electrocardiography and calculated as the average percentage of the heart rate reserve capacity (((heart rate during work – resting heart rate) / (maximum heart rate − resting heart rate)) * 100) during the day.

**Results:**

Using linear regression, significant but weak associations (*β* < 0.23) were observed between perceived exertion and (1) high muscle activity (>60 % of MVC) of the neck muscles and (2) inactivity (<1 % of MVC) of the thigh muscles and (3) cardiovascular load, respectively. Using logistic regression, perceived exertion ≥4 (high exertion), referencing <4 (low-to-moderate exertion), was related to high activity of the trapezius muscle [OR 18 (95 % CI 2–143)], i.e., the odds for experiencing high exertion during work increased 18-fold for each percentage increase in time above 60 % MVC.

**Conclusions:**

During a full working day among blue-collar workers with lifting tasks, high neck muscle activity increases the odds for experiencing high perceived physical exertion. Perceived exertion of at least 4 on the BORG CR10 scale appears to be a good indicator that high muscular loading occurs.

**Electronic supplementary material:**

The online version of this article (doi:10.1007/s00421-013-2782-9) contains supplementary material, which is available to authorized users.

## Introduction

To tackle work-related musculoskeletal problems due to lifting, the Danish Work environment Authority currently uses a model for supervision that primarily focuses on the weight of the load and the perpendicular distance from the center of gravity. The model also takes several other factors into account such as duration, frequency and the shape of the load. While the model in many respects is purposeful shortcomings are also present. One shortcoming is that the model does not take into account individual variations in physical capacity. Obviously, all else equal, a strong person will, in relative terms, use less muscle force to manually lift 20 kg and experience less physical exertion while performing the lift than a weaker person. Accordingly, perceived physical exertion seems to reflect the balance between physical work demands and physical capacity of the individual. Perceived exertion during work is a risk factor for development of musculoskeletal disorders (Andersen et al. 2012a) and sickness absence in certain occupations (Andersen et al. 2012b).

Studies performed in controlled laboratory settings have demonstrated a close relationship between perceived physical exertion and work demands expressed as a percentage of the individual physical capacity. This has been observed both in terms of objectively assessed cardiovascular (Borg 1982; Scherr et al. 2012) and muscular work load (Andersen et al. 2010; Fontes et al. 2010). These observations are interesting because they imply that ratings of perceived exertion could be used to further qualify the existing lifting model. However, only few studies have examined the associations between perceived physical exertion and relative physical workload in workplace settings (Balogh et al. 2004; Village et al. 2005). These workplace studies showed show weak associations between physical exertion and relative physical workload, showing that results from controlled laboratory settings may not necessarily be transferred to the workplace.

There are potentially several reasons for the observed discrepancies between laboratory studies and workplace studies. For example, heart rate monitoring of cardiovascular load provide information on whole body energy expenditure, whereas muscular workload measured by electromyography is associated with local muscular workload. Accordingly, a strenuous workday with separated bursts of high-intensity muscle contractions may have little effect on the average cardiovascular load, but still have significant impact on the perceived physical exertion due to local muscular exertion. Furthermore, hours of repetitive low-to-moderate intensity muscle contractions may affect the average cardiovascular load as well as the perceived physical exertion. Hence, the relative load and the resulting perceived exertion seems highly dependent on the type, intensity and duration of the work performed. As a consequence, the duration and differential tasks of a workday, as opposed to the controlled laboratory tasks, may compromise the association between the objectively assessed workload (cardiovascular and muscular) and the perceived exertion.

Furthermore, as direct measurements are very resource demanding the number of measurements is often reduced to a minimum that may compromise the validity of the measurement. As opposed to direct measurements self-reports of perceived exertion can be applied for a low-cost making it a relevant tool for large workplace surveys. In any event, if perceived exertion in relation to lifting is to be used as a general practical tool in ergonomic supervision situations there is a need to improve the knowledge base by collecting and analyzing individual reports of perceived physical exertion and objectively assessed physical workload (muscular and cardiovascular) during actual work. For this reason, the present study was designed to investigate the association between perceived exertion and objectively assessed muscular and cardiovascular load during a full working day among a broad category of blue-collar workers with lifting tasks.

## Materials and methods

### Participants

**Fig. 1:**
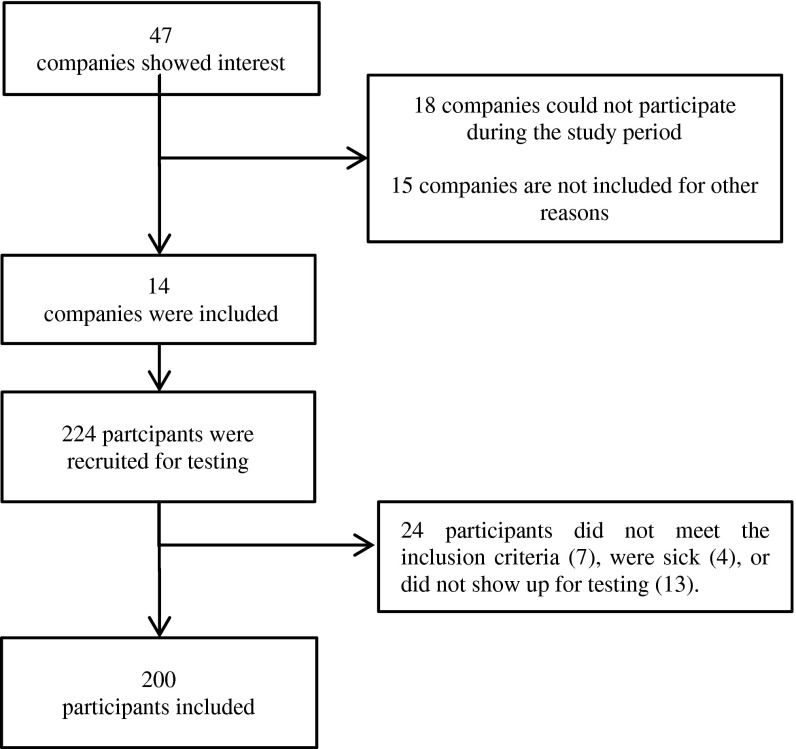
Flowchart of number of companies and participants in the study

A total of 200 employees (159 men and 41 women) with daily manual lifting tasks from 14 different blue-collar companies participated in the study. The participant’s gender and age distribution reflected the gender and age distribution at the workplaces where the investigation occurred (Tables 1, 2). The identification of participants was made in cooperation with the Confederation of Danish Employers, the Confederation of Danish Industry, the Danish Chamber of Commerce and the Danish Construction. Inclusion criteria were employees from companies where manual lifting (not person transfers) was part of the daily work. Exclusion criteria were, disc prolapse, hypertension above 160/100 mmHg, or other serious chronic diseases. Seven of the recruited participants were excluded by these criteria (see flowchart in Fig. 1).

**Table 1:** Age and gender distribution

	<40 years	40–50 years	>50 years	Total
Females	13 (32 %)	14 (34 %)	14 (34 %)	41
Males	71 (45 %)	50 (31 %)	38 (24 %)	159
Total	84	64	52	200

Number of individuals (percentage in parenthesis)

**Tabel 2:** Job types from the 14 companies (*N* = 200)

Company no.	Job type	*N*
1	Machine operator	16
2	Meat cutters	21
3	Postal workers	18
4	Postal-/warehouse workers	21
5	Construction workers	9
6	Brewery workers	5
7	Mechanics	4
8	Meat cutters	16
9	Firefighters	5
10	Gardeners/construction workers	11
11	Construction workers	12
12	Construction workers	27
13	Construction workers	27
14	Shop clerk/warehouse workers	8

All participants were informed about the purpose and content of the survey and gave written informed consent to participate in the study which conformed to The Declaration of Helsinki, and was approved by the Local Ethical Committee (H-3-2010-062).

### Experimental procedure

Participants were asked to present at the worksite for testing 40 min before work, and again in the middle of the workday and after work. All tests were shown and explained and performed discreetly in a quiet room. The strenuous physical tests (strength and cardiovascular fitness) were performed at the end of the workday to avoid fatigue from these tests to influence the findings during the workday.

### Assessment of physical capacity

#### Maximal voluntary isometric contraction (MVC)

Knee extensor, shoulder elevation and back extensor MVCs were performed at the end of the workday to measure maximal strength and maximal muscle activity. Isometric MVC ramp contractions (3-s duration) were performed according to standardized procedures during all MVCs (Andersen et al. 2008b; Jakobsen et al. 2011). During the shoulder and knee extensor MVC, the participants were seated in a specially designed chair adjustable in height ensuring that the subject’s feet had no contact with the floor. Maximal knee extensor strength was measured during seated static knee extensions (knee angle, 90° and hip angle, 90°). The subject was instructed to maximally push against a strap fixated at the ankle 2 fingers above the medial malleolus and horizontally connected to a dynamometer while holding a firm grip on the side of the chair. Shoulder elevation MVC was measured during seated shoulder elevations. Two dynamometers, fixated on a rack, were placed on the medial part of the right and left upper trapezius muscle. The subject was instructed to maximally elevate the shoulders. During lower back extensor MVC, the subject was standing in an upright position with a strap around the shoulders at the level of insertion of the deltoid muscle. The strap was horizontally connected to a strain gauge dynamometer. The subject was facing the dynamometer with the pelvis against a plate placed with the upper edge aligned with the subject’s iliac crest while maximally extending the back.

#### Cardiovascular fitness

Cardiovascular fitness (*V*O_2max_) was estimated from a maximal cycle ergometer test (Watt-max test) (Andersen 1995). This test has been shown to be both valid to direct measurements of oxygen uptake (Andersen 1995) and highly reliable over time (test–retest; ICC = 0.90). Male and female participants started the test with a workload of, respectively, 105 and 70 W on a Monark cycle ergometer (model 874E, Monark AB, Stockholm, Sweden) cycling with a cadence of 70 rpm. An additional 35 W was added every 2 min until the test person was unable to maintain the pedal rate of 70 rpm. The final workload (Watts) and the duration on the last load level (s) specified the maximum power output (MPO (W) = final workload − 35 + (35 × duration on the last load level/120)) from which maximal oxygen uptake (*V*O_2max_) was estimated (*V*O_2max_ = 0.16 + (0.0117 × MPO) (Andersen 1995) and divided by body weight to determine the maximal oxygen uptake (ml O_2_ min^−1^ kg^−1^).

### Assessment of muscular and cardiovascular workload

#### Electrocardiography (ECG) and electromyography (EMG) recording

ECG and EMG signals were recorded from the heart and from three muscles on the leg (vastus lateralis), lower back (erector spinae) and shoulder (trapezius descendens), respectively. The electrodes were placed on the side (right/left) determined by their dominant hand. A bipolar surface EMG configuration (White Sensor, Ambu A/S, Ballerup, Denmark) and an inter-electrode distance of 2 cm were used (Andersen et al. 2006, 2008a, c; Jakobsen et al. 2011, 2012). Before affixing the electrodes, the skin of the respective area was prepared with scrubbing gel (Acqua gel, Meditec, Parma, Italy) to effectively lower the impedance to less than 10 kΩ. Electrode placements followed SENIAM recommendations (http://www.seniam.org). The electrodes were fixated with tape (Fixomull stretch) and connected through thin shielded cables to a datalogger (Nexus10, Mind Media, Netherlands) that was placed in a flexible belt to ensure mobility for the worker throughout the working day.

#### Electrocardiography and electromyography analysis

EMG activity from the vastus lateralis, erector spinae, upper trapezius and ECG activity (heart rate) were sampled at 1,024 Hz using Nexus10 data loggers (Mind Media, Netherlands). Data filtering and data analysis were performed with custom-made Matlab programs (MathWorks).

All EMG and ECG data were digitally filtered according to the linear envelope method (Winter 1990); a highpass filtering (EMG, 10 Hz; ECG, 4 Hz cuttoff, 4th order Butterworth filter) followed by a full-wave rectification and lastly a lowpass filtering (EMG, 2.2 Hz; ECG, 2 Hz cuttoff, 4th order Butterworth filter). The filtered EMG signals were normalized with respect to maximal muscle activity obtained during the MVCs. In order to determine heart rate (HR), each peak of the filtered ECG signal was identified.

The following EMG parameters were calculated to determine the relative muscular load: mean value of the normalized EMG signal and the percentage of the working day (excluding lunch) where the signal was less than 1 % (inactivity) and over 20 % (moderate activity), 40 % (moderate-to-high activity) and 60 % (high activity) of the normalized EMG signal, respectively. The cardiovascular load was defined as the average percentage of the heart rate reserve capacity (((heart rate during work – resting heart rate) / (maximum heart rate − resting heart rate)) * 100) during the day. The maximum heart rate was measured during the Watt-max test, whereas the resting heart rate was measured prior to work after the subjects were given information about the purpose of the study.

### Assessment of perceived exertion

The participants were trained to use the Borg CR-10 scale during a standardized lifting task prior to the work (data not shown). During the survey the participant’s anonymity was ensured so no colleagues, managers or others were able to influence the responses.

#### Perceived exertion

The Borg CR10 scale (Borg 1998) was used to collect ratings of perceived exertion on three times during the working day. The scale had 15 steps, some of which were verbally anchored, and higher values indicated greater perceived exertion. The question was administered via a computer to the participants and was phrased “rate your perceived exertion for the past 2 h”. The respondents had to answer this question three times: in the morning prior to work start, at lunch and at the end of the working day. To reduce the variation in self-reports of perceived exertion, we used the average value of two measurements obtained at midday and at the end of the working day.

In the present study, both the continuous score (0–10) and a categorical classification of scores were used. The categorical classification was based on the wording in the response scale. This method was preferred over other forms of categorization (e.g., median or quartile splits) since it increases subsequent interpretability. Three groups were formed in relation to their perceived exertion during work:

- Weak exertion (Borg ≤ 2),
- Moderate exertion (2 < Borg < 4), or
- High exertion (Borg ≥ 4).

In the following, we term these as exertion groups 1, 2 and 3, respectively.

### Statistical analysis

A general linear model, PROC GLM of SAS version 9.2., adjusted for age and gender was used to describe overall differences in the objectively assessed parameters between the three exertion groups scoring weak (≤2), moderate (2 < Borg < 4), or high (Borg ≥ 4) perceived exertion during the day (Table 3).

**Tabel 3:** Anthropometric, physical capacity, muscle activity and cardiovascular load data of the participants divided into three groups based on their perceived exertion (BORG CR10 scale)

	Group 1	Group 2	Group 3	*p* value	
	Low (0.0–2.0)	Moderate (2.1–3.9)	High (4.0–10)	1 vs. 3	2 vs. 3
Number of participants (% of all participants)	88 (44 %)	87 (43 %)	25 (13 %)		
% of males	78 %	83 %	72 %		
Age	41 (11)	44 (10)	34 (12)	**	**
Height (cm)	177 (9)	176 (9)	176 (8)		
Weight (kg)	80 (15)	79 (14)	78 (14)		
BMI (kg/m^−2^)	26 (4)	25 (3)	25 (3)		
Physical capacity					
Muscle strength					
Leg (N m)	139 (40)	144 (45)	129 (48)		
Back (N m)	174 (58)	178 (55)	173 (66)		
Neck/shoulder (N m)	125 (45)	124 (45)	120 (51)		
Hand grip (kg)	49 (12)	49 (11)	50 (14)		
Cardiovascular fitness (ml O_2_ kg^−1^ min^−1^)	33 (8)	32 (8)	31 (6)		
Relative intensity during work					
Inactivity in muscles (% of work time < 1 % max EMG)					
Leg	64 (16)	59 (15)	58 (14)	*	
Back	22 (21)	20 (20)	21 (20)		
Neck/shoulder	15 (10)	17 (13)	15 (9)		
Muscle activity with moderate intensity (% of work time > 20 % max EMG)					
Leg	2.5 (3.2)	2.5 (3.5)	2.1 (2)		
Back	3.6 (6.5)	4.5 (7.4)	3.1 (3.8)		
Neck/shoulder	4.2 (4.4)	4.2 (4.2)	5.7 (8.8)		
Muscle activity with moderate–high intensity (% of work time > 40 % max EMG)					
Leg	0.45 (0.8)	0.40 (0.8)	0.42 (0.6)		
Back	0.21 (0.4)	0.39 (1)	0.21 (0.3)		
Neck/shoulder	0.45 (0.6)	0.47 (0.6)	0.93 (1.9)	*	*
Muscle activity with high intensity (% of work time > 60 % max EMG)					
Leg	0.09 (0.18)	0.10 (0.25)	0.13 (0.25)		
Back	0.03 (0.09)	0.05 (0.20)	0.04 (0.09)		
Neck/shoulder	0.07 (0.09)	0.08 (0.11)	0.23 (0.48)	**	**
Cardiovascular load during the working day (% of heart rate reserve capacity)	18 (9)	20 (8)	24 (6)	**	*

Values are presented as mean (SD)

* *p* < 0.05; ** *p* < 0.01. *F* test adjusted for age and sex

Both multi-variate linear regression (Proc Reg of SAS) (Table 4) and logistic regressions (Proc Logistic of SAS) (Table 5) was used to test the associations between perceived exertion and objectively assessed muscular and cardiovascular load. In Model 1, the independent variables were sex, age, BMI and muscle activity (high activity in the neck/shoulder and inactivity in the legs). In Model 2, the independent variables were sex, age, BMI and cardiovascular load. Finally, in Model 3, the independent variables were sex, age, BMI and muscle activity and cardiovascular load. The reason for using both linear and logistic regression was to explore whether the associations appeared linear or whether a threshold existed.

**Table 4:** Associations (multiple linear regression, standardized beta-values) between objectively assessed cardiovascular (% of heart rate reserve capacity) and muscular load (EMG) and perceived exertion during work

	Inactivity in the leg muscles (% of work time < 1 % max EMG)	Neck and shoulder muscle activity with high intensity (% of work time > 60 % max EMG)	Cardiovascular load during the working day (% of heart rate reserve capacity)
Model 1	0.16	0.23*	
Model 2			0.19*
Model 3	0.14	0.22*	0.08

Model 1: Independent variables were sex, age, BMI and muscle activity (high activity in the neck/shoulder and inactivity in the legs)

Model 2: Independent variables were sex, age, BMI and cardiovascular load

Model 3: Model 1 + 2

* *p* < 0.05

**Table 5:** Logistic regression odds ratios for perceived exertion during work

	Inactivity in the leg muscles (% of work time < 1 % max EMG)	Neck and shoulder muscle activity with high intensity (% of work time > 60 % max EMG)	Cardiovascular load during the working day (% of heart rate reserve capacity)
Model 1	1.03 (0.99–1.06)	18.1 (2.3–143.3)*	
Model 2			1.09 (1.02–1.15)*
Model 3	1.02 (0.98–1.06)	18.8 (2.3–153.0)*	1.07 (0.99–1.15)

Model 1: independent variables were sex, age, BMI and muscle activity (high activity in the neck/shoulder and inactivity in the legs)

Model 2: independent variables were sex, age, BMI and cardiovascular load

Model 3: Model 1 + 2

* *p* < 0.05

Descriptive data are reported as least square means (SD). Regression coefficients from the linear regression are reported as standardized betas, and odds ratios from the logistic regression; *p* values ≤0.05 were considered statistically significant.

## Results

### Perceived exertion during work

**Fig. 2:**
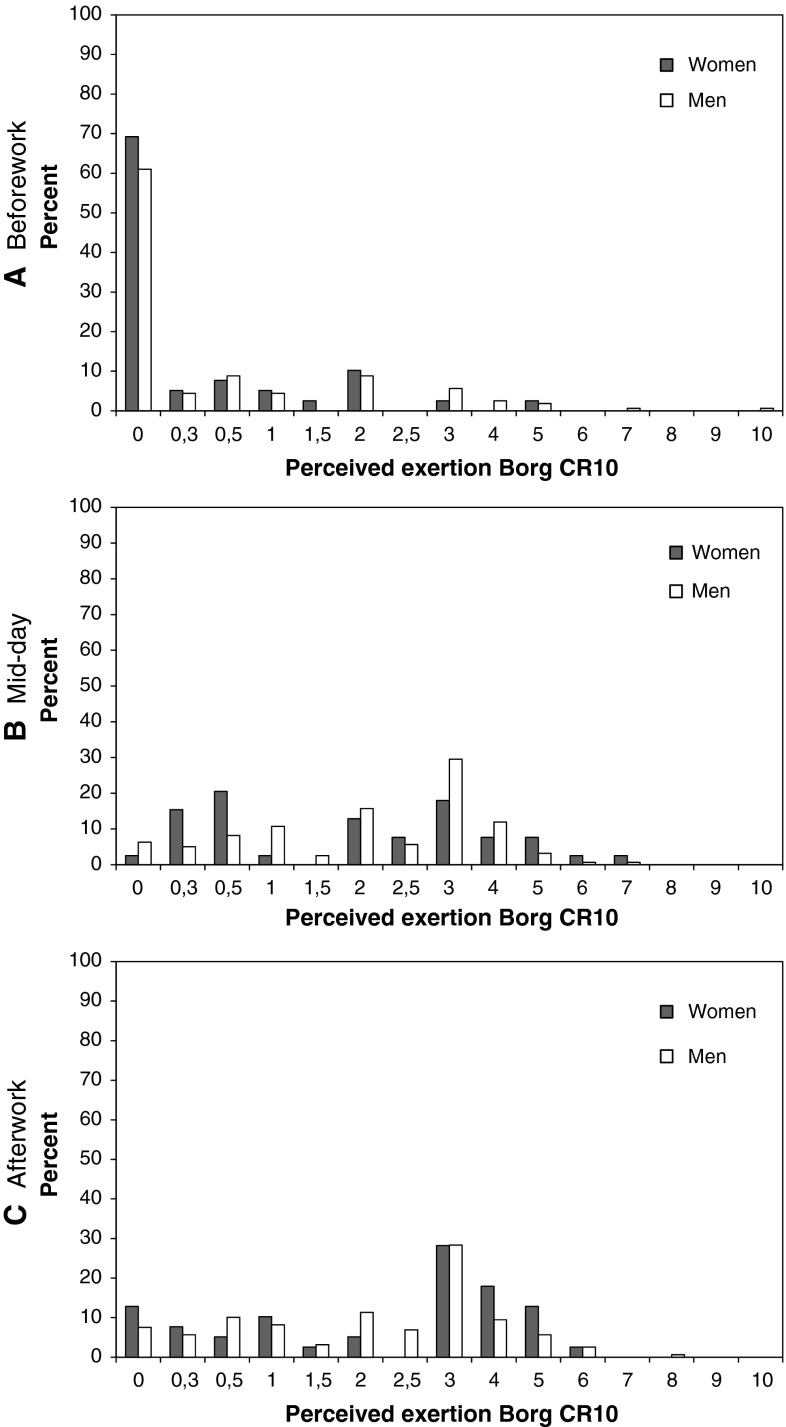
Frequency distribution of perceived exertion rated for the past 2 h before work (**a** mean Borg 0.7), at midday (**b** mean Borg 2.2) and at the end of the working day (**c** mean Borg 2.4) for 200 blue-collar workers (women *n* = 41 and men *n* = 159) with lifting tasks

Figure 2 shows the frequency distribution of perceived exertion rated for the past 2 h before work [median 0.0 (25th percentile 0.0, 75th percentile 0.5)], at midday [median 2.5 (25th percentile 1.0, 75th percentile 3.0)] and after the working day [median 2.5 (25th percentile 1.0, 75th percentile: 3.0)]. There were significant differences (*p* < 0.001) between perceived exertion obtained before work (0.7 ± 0.10) versus at midday (2.2 ± 0.10) and after the working day (2.4 ± 0.11). There was no difference (*p* > 0.05) between perceived exertion obtained at midday and after the working day.

### Exertion group comparison adjusted for age and gender

Table 3 shows differences of the main variables between the three exertion groups when adjusted for age and gender. During the workday, group 3 (i.e., those perceiving high physical exertion during work) demonstrated higher cardiovascular load (*p* = 0.004 and *p* = 0.032, respectively) (% of the heart rate reserve capacity), longer time with moderate to high (% of work time > 40 % max EMG) (*p* = 0.021 and *p* = 0.041, respectively) and high (% of work time > 60 % max EMG) (*p* < 0.001 and *p* = 0.002) muscle activity in the neck/shoulder muscles compared to group 1 and group 2, respectively. Compared to group 3 the participants of group 1 demonstrated a greater percentage of the workday with inactivity in the legs (*p* = 0.020).

There were no differences in gender, height, weight, BMI, muscle strength and cardiovascular fitness between the three exertion groups. However, the participants reporting high perceived exertion (exertion group 3) were characterized by being younger (*p* = 0.004 and *p* < 0.001, respectively) than those reporting lower perceived exertion during the workday (group 1 and group 2, respectively).

### Multiple linear regression analysis

Table 4 shows the associations between objectively assessed cardiovascular (% of heart rate reserve capacity) and muscular (EMG activity) load and perceived exertion obtained during a full workday. The multiple linear regression analysis demonstrated significant but weak associations between muscle activity (high activity in the neck/shoulder and inactivity in the legs) and perceived exertion during the workday (Model 1) and cardiovascular load and perceived exertion during the workday (Model 2). However, in the fully adjusted model including both muscle activity and cardiovascular load (Model 3), only the muscle activity of the neck/shoulder muscles was significantly related (*β* = 0.22) to perceived exertion.

### Logistic regression analysis

The results of Table 5 indicate that a threshold of perceived exertion exists for high levels of neck/shoulder (trapezius) muscle activity. In the logistic regression analysis, we dichotomized the Borg scale calculated the probability of experiencing “high” exertion (Borg ≥ 4) during the work day compared with “low or moderate” exertion (Borg < 4). Table 5 lists the odds ratios [OR (95 % CI)], and statistically significant findings are highlighted with an asterisk. The present OR should be read as follows; an OR of 18 for the neck/shoulder muscle activity increases the likelihood of experiencing “high” exertion by a factor of 18 for every percentage point increase in the total duration of the workday with high muscle activity in the neck/shoulders. With regard to cardiovascular load, the OR was 1.09 meaning that for every percentage point increase in cardiovascular load (% of heart rate reserve capacity) during the workday the likelihood of experiencing “high” exertion was increased by a factor of 1.09.

Because the incidence of pain has been associated with misclassification of self-rated exposure (Hansson et al. 2001; Balogh et al. 2004), we tested the data, on an exploratory basis, with participants with low levels of musculoskeletal pain only. Nevertheless, excluding participants with moderate and high back and neck/shoulder pain (VAS < 3; Gr1: *n* = 49, Gr2: *n* = 38, Gr3: *n* = 5) did not change the overall results as observed in Table 1 and in the logistic and multiple linear regression analysis.

## Discussion

The present study investigated the association between perceived exertion and objectively assessed muscular and cardiovascular load during a full working day in jobs characterized by lifting tasks. Logistic and linear multiple regression analysis demonstrated significant associations between perceived exertion and objectively assessed cardiovascular and muscular load. Particularly, muscular load measured as the percentage of the working day with high neck/shoulder muscle activity was associated with perceived exertion. The logistic regression analysis indicates that a threshold exists when experiencing high (Borg ≥ 4) perceived exertion.

Our observations show that many participants reported a moderate perceived exertion during work. These reported levels of perceived exertion are in fair agreement with the reported levels of perceived exertion among Danish blue-collar workers that were engaged in heavy construction work (Persson et al. 2006). Accordingly, the participants seem to be fairly good representatives for people in strenuous work. However, it is also clear that the perceived exertion ratings are skewed to the lower end (cf. Figure 2).

When dividing the participants into three perceived exertion groups that grouped the answers in relation to the meaning of the underlying response scale (“low”, “moderate” and “high”), we observed significant age and gender-adjusted group differences in objectively assessed cardiovascular and muscular load. Accordingly, the “high” perceived exertion group demonstrated longer periods with moderate and high muscle activity (% of work time > 40 and 60 % max EMG, respectively) and greater cardiovascular load compared to the “low” and “moderate” group. Furthermore, participants experiencing “low” perceived exertion demonstrated longer periods with inactivity in their leg muscles compared with the “high” perceived exertion group. In support of this, the multiple linear regression analysis demonstrated significant, but weak associations between muscle activity (high activity in the neck/shoulder and inactivity in the legs) and perceived exertion and cardiovascular load and perceived exertion assessed during the workday. However, in the fully adjusted linear model including both muscle activity and cardiovascular load, only the muscle activity of the neck/shoulder muscles remained significantly related to perceived exertion. Correspondingly, when dichotomizing the Borg scale into “low or moderate” (Borg < 4) and “high” (Borg ≥ 4) perceived exertion, the logistic regression analysis demonstrated strong association between perceived exertion and high neck/shoulder muscle activity. Notably, this indicates that the associations are non-linear and a threshold exists when experiencing high (Borg ≥ 4) perceived exertion.

On average, the neck muscles were exposed to longer periods of high muscle activation compared to leg and lower back muscles during the workday. As implied by the types of jobs investigated (cf. Table 2), this indicates that the majority of tasks were performed either seated or standing fully erect at an assembly line or table lifting boxes, handling meat, etc. Accordingly, this supports the strong association between high neck muscle activity and perceived exertion and the lack of association between lower back and leg muscle activity and perceived exertion. On the other hand, several of the jobs investigated may have had higher relative loading on muscles that were not examined, i.e., hand- and arm muscles (i.e., in meat cutters and machine operators). As a consequence, this may have weakened the relationship between perceived exertion and the muscular load of the muscles examined. Nevertheless, the current muscles represent large muscle groups where one or several groups to some extent are involved in almost all tasks performed in the present job types. Accordingly, when, i.e., cutting meat using hand and arm muscles, the neck/shoulder muscles are also highly active (Arvidsson et al. 2012) and would inherently contribute to the association between trapezius muscle load and perceived exertion.

In line with the present findings, Balogh et al. 2004 observed comparable low associations between perceived exertion and cardiovascular load in day measurements in cleaners and office workers (Balogh et al. 2004). It may therefore be suggested, that the relative cardiovascular load induced by the investigated jobs was not high enough to have significant impact on perceived exertion.

It may in work situations, be difficult for the individual to distinguish between the lowest levels on the Borg scale, for example very weak, weak and moderate exertion (Borg 1–4), which can increase the risk of misclassifications and thereby bias the findings of our linear correlation analysis. As a consequence, the Borg scale was divided into a “low” (<4) and a “high” (≥4) perceived exertion part in the logistic regression analysis. The odds ratio of 18 observed between perceived exertion and neck/shoulder muscle activity means that the likelihood of experiencing “high” exertion is increased by a factor of 18 for every percentage point (1 percentage point is 4½ min of a 7½ h shift) increase in the total duration of the workday with high muscle activity in the neck/shoulders. Translated into practical guidelines, this roughly means that for every 15 heavy lifts of 1 s with high muscle activation the likelihood of experiencing “high” perceived exertion is doubled. In view of this, the workers in the group that rated “high” perceived exertion (≥4 Borg) worked on average 0.23 % of the day (~7½ h shift) with high neck/shoulder muscle activity (>60 % of max EMG). This corresponds to a total exposure of ~1 min of high muscle activity or in more practical terms roughly 65 heavy lifts at an estimated lift duration of 1 s. Accordingly, as high physical exertion is a risk factor for development of musculoskeletal disorders (Andersen et al. 2012a) long-term sickness absence (Andersen et al. 2012b), the present results may help in the development of better lifting-guidelines for preventing work-related poor health in blue-collar workers.

The incidence of musculoskeletal complaints has been associated with misclassification of self-rated exposure. Several studies have shown that people with musculoskeletal disorders rate their exposure higher than healthy people (Hansson et al. 2001; Balogh et al. 2004). Furthermore, Viikari-Juntura et al. (1996) observed that the relationship between self-reports and hand, neck and shoulder movements was lower for workers with low-back pain than those without pain. Accordingly, it seems that pain has a greater effect on perceived exertion than on the balance between physical capacity and workload that may bias the association between self-report and direct measurement. However, adjusting for self-reported muscle pain in a model with self-reported physical exertion is meaningless, because muscle pain may be both the cause and effect of physical exertion. Thus, if physical exertion leads to muscle pain, adjusting for muscle pain will underestimate the association between perceived exertion and the objectively assessed variables. Another method is to stratify analyses, however, at the risk of hampering generalizability of the findings. On an exploratory basis, when excluding participants with moderate and high pain (VAS > 3 in either body region) only the differences in moderate-to-high and high neck/shoulder muscle activity remained significant between the “high” and moderate and “low” perceived exertion group. Thus, regardless of pain, this highlights the strong association between neck/shoulder muscle activity and perceived exertion in manual strenuous jobs.

As previously indicated, the large variability in job types, tasks and physical capacity of the worker may cause a large variation in perceived exertion. However, this only strengthens the generalizability of our findings. Nevertheless, possible further explanations that may have compromised the association between perceived exertion and the objectively assessed workload may be addressed the complexity and frequency of the question asked (Spielholz et al. 2001; Stock et al. 2005). The participants were asked to rate their average perceived exertion of the past 2 h at midday and after 7½ h of work on a Borg CR10 scale. Although the question seems rather simple, it may be difficult to provide a valid estimate of the average exertion experienced during the past 2 h, indeed if the subject had performed several different tasks. It may be speculated that the association between perceived exertion and objectively assessed workload would have been higher if the frequency of questions were higher during the day or alternatively if the workers could rate their perceived exertion after every task performed. However, because only minimal data collection, e.g., a single question posed, is feasible in a real-world workplace setting, the aim of this study was to investigate whether a minimum of perceived exertion reports obtained during the day could provide valuable evidence of a relationship between objectively assessed physical workload and perceived exertion of a full working day.

There are both strengths and limitations to our study. Because maximal isometric muscle activity and maximal heart rate capacity may be reduced in participants with musculoskeletal disorders (Andersen et al. 2008c, 2009) the present type of EMG and ECG normalization may be a limitations. On the other hand, this procedure has been widely used (Andersen et al. 2006; Jakobsen et al. 2011) and has shown to generate greater test–retest reliability scores compared with non-normalized data (Wilk et al. 1996; Kellis and Baltzopoulos 1996; Rutherford et al. 2001; Alkjaer et al. 2003). Additionally, performing maximal exertion-tests (MVC and *V*O_2max_) after a strenuous day of work may potentially skew the results. Nevertheless, normalizing EMG with maximal EMG values obtained from separate days is not advisable and performing the MVC and *V*O_2max_ test on two separate days was not possible for practical reasons.

Furthermore, an exploratory analysis excluding employees with musculoskeletal pain did not change the results. A strength is the large population size and variability among participants and job types, highlighting the generalizability of the study results.

## Conclusion

In the present study using objective measures during a full working day among blue-collar workers with lifting tasks, high trapezius muscle activity increases the odds for experiencing high perceived physical exertion. Perceived exertion of at least 4 on the BORG CR10 scale appears to be a good indicator that high muscular loading occurs during the workday. This knowledge may be useful when supervising the intensity of physical workload for individual workers.

## Electronic supplementary material

Below is the link to the electronic supplementary material.

Supplementary material 1 (DOC 28 kb)
