# Supplementary material for: Is Borg’s perceived exertion scale a useful indicator of muscular and cardiovascular load in blue-collar workers with lifting tasks? A cross-sectional workplace study
Source: Eur J Appl Physiol. 2013 Dec 13;114(2):425–34. doi: 10.1007/s00421-013-2782-9 (PMC3895215; doi:10.1007/s00421-013-2782-9)
Supplement: Supplementary file 1 — Supplementary material 1 (DOC 28 kb) [file 421_2013_2782_MOESM1_ESM.doc]

Borg CR10 scale

Please rate your perceived exertion obtained for the past two hours.

***’0’: nothing at all***

***’10’: the maximal exertion that you can imagine if you use all your effort***

**Number Perceived exertion**

0 Nothing at all

0.3

- 1. Just noticeable

1 Very light

1.5

2 Light

2.5

3 Moderate

4 Somewhat heavy

1. Heavy

6

7 Very heavy

8

9

1. Maximal

*Borg CR-10 skala*
